# Nitrogen Balance after the Administration of a Prolonged-Release Protein Substitute for Phenylketonuria as a Single Dose in Healthy Volunteers

**DOI:** 10.3390/nu13093189

**Published:** 2021-09-14

**Authors:** Mika Scheinin, Jouni Junnila, Giorgio Reiner, Anita MacDonald, Ania C. Muntau

**Affiliations:** 1 CRST Oy, Itäinen Pitkäkatu 4B, FI-20520 Turku, Finland; 2 Institute of Biomedicine, University of Turku and TYKSLAB, Turku University Hospital, Kiinamyllynkatu 4-8, FI-20520 Turku, Finland; 3 Oy 4Pharma Ltd., Arkadiankatu 7, FI-00100 Helsinki, Finland; jouni.junnila@estimates.fi; 4 EstiMates Oy, Kunnallissairaalantie 56 as 1, FI-20810 Turku, Finland; 5 APR Applied Pharma Research SA, Via Corti 5, CH-6828 Balerna, Switzerland; giorgio.reiner@apr.ch; 6 Dietetic Department, Birmingham Women’s and Children’s Hospital NHS Foundation Trust, Birmingham B4 6NH, UK; anita.macdonald@nhs.net; 7 University Medical Center Hamburg Eppendorf, University Children’s Hospital, Martinistrasse 52, D-20246 Hamburg, Germany; muntau@uke.de

**Keywords:** phenylketonuria, nitrogen balance, amino acid catabolism, blood urea nitrogen, prolonged release

## Abstract

Nitrogen balance is the difference between nitrogen excreted as urea and nitrogen ingested, mainly in proteins. Increased circulating concentrations of amino acids (AA) in the bloodstream are usually associated with proportional increases in the production and excretion of urea. Previously, we reported results from a randomized, controlled, single-dose, crossover trial in healthy adult volunteers (n = 30) (Trial Registration: ISRCTN11016729), in which a Test product (prolonged-release AA mixture formulated with Physiomimic Technology™ (PT™)) significantly slowed down the release and reduced the peak plasma concentrations of essential AAs compared with a free AA mixture (Reference product) while maintaining essential AA bioavailability. Here, we report an assessment of the nitrogen balance from the same study. The amount of nitrogen contained in plasma AAs, levels of blood urea nitrogen (BUN) (*p* < 0.0001) and changes in BUN (*p* < 0.0001) were smaller after the Test product compared with the Reference product. These findings suggest that the production of urea in proportion to systemic AA availability was significantly smaller after the administration of the Test product compared with the Reference product and that the test product conferred the increased utilization of AAs for protein synthesis and reduced their oxidation and conversion to urea. In the clinical setting, it is possible that the effects of PT™ observed on the disposition of free AAs in this study may translate to health benefits in terms of physiological body composition and growth if used for the treatment of subjects with phenylketonuria (PKU). Further investigation in patients with PKU is warranted.

## 1. Introduction

Phenylketonuria (PKU) is the most common inherited disease of amino acid (AA) metabolism, with a global prevalence of 0.3–38.1 per 100,000 newborns [1]. For more than half a century, patients with PKU have been treated with a phenylalanine-restricted diet combined with phenylalanine-free AA mixtures to compensate for the low intake of natural proteins. However, the administration of free AAs produces several metabolic imbalances not observed with equilibrated diets consisting of food containing intact natural proteins. A recent systematic review and meta-analysis reported that even with advances in dietary treatments, ‘optimal’ growth outcomes are not always attained in children with PKU on a Phe-restricted diet. In contrast, growth is similar to reference populations in children with mild hyperphenylalaninemia not requiring dietary restriction [2]. The unsavoury taste of products containing free AAs often leads to a low adherence to dietary management and may significantly increase the burden of the disease [3,4,5,6]. Despite several improvements in recent years, a taste and odour-free protein substitute with the properties of natural protein still remains the main feature in order to guarantee an optimal physiological function and patient tolerance [4].

**Figure 1:**
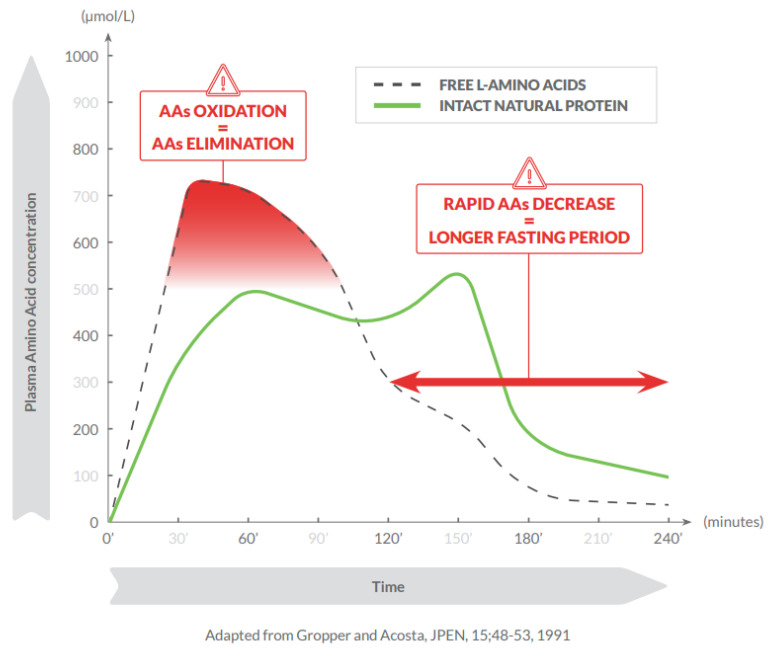
Consumption of free amino acids results in higher plasma concentrations, earlier absorption peak, and steeper blood concentration reductions compared with intact natural proteins.

The more rapid absorption of free AAs compared with AAs from intact proteins is associated with a less efficient utilization, early oxidation and effects on insulin release, glycaemic control, and endocrine regulation [7,8]. The intake of free AAs results in higher plasma concentrations, earlier absorption peaks, and steeper blood concentration reductions compared with the intake of intact natural proteins [9] (Figure 1).

AAs serve many functions in the body. Being the only source of nitrogen for mammals, AA-derived nitrogen is pivotal for synthetizing precursors of major energy molecules (i.e., ATP, ADP, IMP) and/or nucleic acids (i.e., DNA/RNA), and/or to produce compounds that can regulate major biochemical signalling pathways, such as nitric oxide [10]. Moreover, the deamination of AAs released from skeletal muscle and/or circulating dietary proteins generates a carbon skeleton rich in oxygen and hydrogen suitable for the subsequent biochemical transformation. This carbon skeleton can be used by the liver to produce glucose, through gluconeogenesis, and other important molecules, such as lipids. The AA-derived carbon skeleton is also relevant in producing intermediates fuelling the Krebs cycle that are, thereafter, transformed into energy and/or other metabolic intermediates. Therefore, AAs may be converted into energy, carbohydrates, lipids, and biochemical intermediates, dependent on the body’s metabolic demands.

β oxidation, which is mostly mitochondrial, reduces the ratio of ATP/available oxygen, and obliges large amounts of essential AAs (EAAs) to be used as intermediates of the Krebs cycle. Such a metabolic shift is one of the main alterations leading to an imbalance between nitrogen demand and nitrogen intake observed in patients with chronic altered metabolic conditions, and is measured as the nitrogen balance [10].

Urea is the end product of protein catabolism in the liver, and the association between plasma AA concentrations and urea production is almost linear, i.e., increasing circulating concentrations of AAs result in proportional increases in the production and plasma concentration of urea [11]. Blood urea nitrogen (BUN) is a clinically employed indicator of nitrogen harboured by urea.

Mönch [12] reported that bolus administration of free AAs increases the amount of nitrogen excreted into urine, when the rapid increase in circulating AAs exceeds the capacity of anabolic processes to incorporate them into nascent proteins (protein synthesis). Similarly, when young healthy subjects were fed with ‘slow’ proteins (e.g., casein), protein retention was greater than in subjects fed with ‘fast’ proteins (e.g., whey); i.e., rapid AA uptake was associated with a rapid increase in blood AAs and higher oxidation rates [13,14].

The impact of free AAs on physiological and metabolic balance has prompted the search for nutritional strategies for patients with PKU that would closely match physiological circumstances [15,16]. Physiomimic Technology™ (PT™) is a pharmaceutical process that results in small granules coated with functional additives—ethylcellulose and sodium alginate—that allow the gradual release of their contents in the small intestine. PT™ modifies the release and absorption of AAs, while masking their taste and odour, with positive effects on the typically unpleasant aftertaste of traditional AA formulations. Preliminary evidence for use of this technology was obtained from a porcine model, where the application of PT™ to free AA mixtures reduced the peak blood concentration (C_max_) by 18%, while maintaining a similar overall increase in plasma AAs [17].

As reported previously, we conducted a study in healthy adult volunteers to determine the effects on plasma AA profiles of a prolonged-release AA mixture formulated with PT™, comparing it with an immediate-release formulation of the same AA mixture, a commercially available free AA mixture and a natural intact protein, casein [18]. The study results showed that an AA mixture formulated with PT™ significantly prolonged the release of AAs, lowered peak EAA levels in plasma, and maintained an equivalent overall increase in plasma EAAs [18]. Here, we report more results from the same study, now comparing nitrogen balance after the administration of the PT™-formulated Test product and a Reference product containing free AAs.

## 2. Materials and Methods

### 2.1. Study Design

All study participants provided written informed consent. The study was conducted at CRST Oy in Turku, Finland, and was approved by the Ethics Committee of the Hospital District of Southwest Finland, Turku, Finland; ref: 78/1801/2017. Trial registration: ISRCTN11016729 [18]. Briefly, in this randomized, controlled, single-blind, crossover trial, the kinetic profiles of different AA preparations were assessed in 30 healthy volunteers (15 male, 15 female) aged between 18 and 45 years with body weight between 55 and 85 kg and body mass index ≤ 30 kg/m^2^.

The Test product was a phenylalanine-free AA formulation, engineered with the PT™, containing 17 AAs, vitamins, minerals, other nutrients, ethylcellulose, and sodium alginate as food additives. The Reference product was a phenylalanine-free AA formulation with the same qualitative and quantitative composition as the Test product (in terms of AAs, vitamins, minerals, other nutrients, ethylcellulose, and sodium alginate). The only difference was that no coating layer was used.

**Figure 2:**
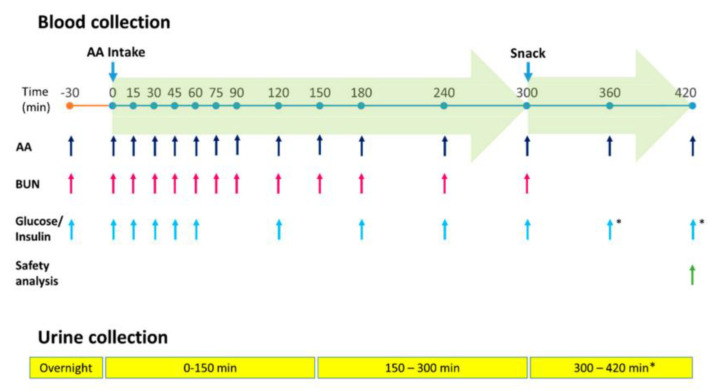
Administration, sample collection, and analysis schedule. AA, amino acids; BUN, blood urea nitrogen. * for safety assessment only.

The Test product and Reference product were administered in single doses (0.40 g AA/kg body weight) at time 0 (Figure 2). This single dose represented 1 of the 3 doses necessary to cover the daily AA requirements for adults with PKU. In this crossover study, the days on which the healthy volunteers received the study products were separated by a 9–14-day wash-out period. On each test day, venous blood samples and urine samples were obtained at regular intervals according to the analysis schedule.

### 2.2. Statistical Analysis

All results are described as mean and standard deviation. The nitrogen concentration data (separately for AA and BUN) were analysed with repeated measures analysis of variance (RMANOVA) models, where product sequence, product, timepoint, and the two-way interactions of sequence*timepoint and product*timepoint were used as fixed effects and subject within sequence and residual error term as random effects. The balance between nitrogen concentrations from AA administration in blood and BUN was analysed with a similar RMANOVA model, using the difference between blood and BUN nitrogen (within product) as the response variable.

The quantity of nitrogen (both in blood and BUN, calculated from the areas under the curve from 0 (baseline) to 300 min (AUC_0__–__300_) contained in AAs and in BUN) was analysed with a linear mixed effect model on log-transformed data. The statistical model included sequence and product as fixed effects, and subject within sequence and residual error term as random effects. The 95% confidence interval (CI) for the nitrogen quantity was calculated from the model for equivalence evaluation. CI estimates were converted by anti-log transformation to obtain ratios of geometric least square means.

## 3. Results

Thirty subjects successfully completed the intervention with the Reference product, and twenty-eight subjects successfully completed the intervention with the Test product.

### 3.1. Blood AA and Nitrogen Concentrations

Total plasma AA concentrations for the Test and Reference products from baseline to 300 min after a dose intake and the differences in concentrations (mmol/L) of the Test product minus the Reference product (Delta AA) were determined (Table 1). For the time points from 15 min to 150 min, the concentrations of AAs were lower after the administration of the Test product compared with the Reference product. For the time points from 180 min to 300 min, the concentrations of AAs were higher after the administration of the Test product than the Reference product. Nitrogen concentrations and the nitrogen concentration differences (Delta) for the Test and Reference Products were calculated considering that some AAs contain two nitrogen atoms (such as glutamine, lysine, and tryptophan) or three nitrogen atoms (such as arginine and histidine). The Delta concentration of each AA and the Delta nitrogen concentrations were calculated for each time point, using a conversion factor that accounted for the different numbers of nitrogen atoms in each AA.

### 3.2. BUN and Nitrogen Concentrations

Urea is a waste product that is formed in the liver when the body breaks down AAs; BUN reflects the nitrogen content in urea (molecular weight 28). The concentrations of BUN and urea are equal when expressed as mmol/L because both entities contain two nitrogen atoms.

Total plasma BUN concentrations for the Test and Reference products from baseline to 300 min and the differences in concentrations (mmol/L) of the Test product minus the Reference product (Delta BUN) were determined (Table 2). The concentration of BUN was lower at all time points following the administration of the Test product compared with the Reference product. To calculate the differences in the nitrogen concentration (mmol/L) of the Test product minus the Reference product (Delta nitrogen), the Delta BUN (mmol/L) had to be multiplied by two (since one molecule of urea contains two nitrogen atoms).

BUN concentrations and, consequently, the urea-bound nitrogen concentrations were consistently lower after the Test product than after the Reference product, indicating a lesser oxidation of AAs after the intake of the Test product. This overall difference between the products was statistically highly significant (*p* < 0.0001).

### 3.3. Balance between Nitrogen Concentrations from AA Administration in Blood and BUN

Based on the observations presented in Table 1 and Table 2, it was possible to evaluate the balance between the Delta nitrogen concentrations contained in plasma AAs and in BUN after the administration of the Test and Reference products (Table 3). Delta nitrogen from AAs was negative over the first 120 min after the ingestion of the Test product, becoming positive from 180 min onwards, confirming the slower release and absorption of AAs from the Test product compared with the Reference product. Conversely, Delta nitrogen in BUN remained negative until 300 min, confirming a lower production of waste nitrogen (BUN) after the administration of the Test product compared with the Reference product.

**Figure 3:**
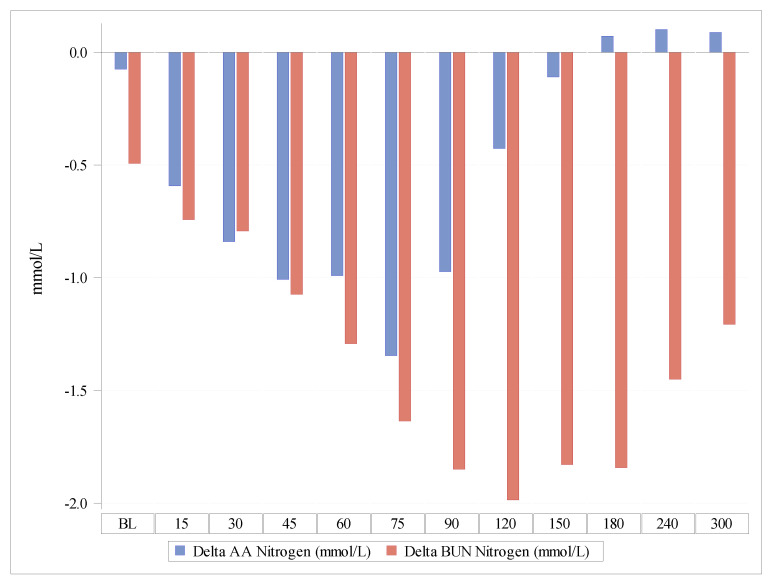
Delta of the nitrogen concentrations contained in plasma amino acids in comparison with Delta nitrogen concentrations as blood urea nitrogen. AAs, amino acids; BUN, blood urea nitrogen.

**Figure 4:**
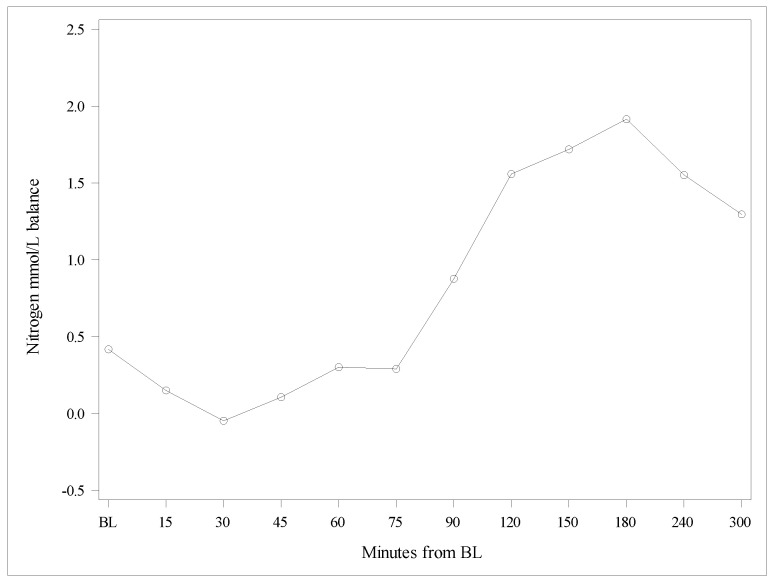
Differences between Deltas of nitrogen concentrations from amino acids, and nitrogen from blood urea nitrogen. BL, baseline.

The Delta of nitrogen concentrations in BUN versus the Delta of nitrogen concentrations contained in plasma AAs increased over time, indicating a lesser oxidation of AAs after the Test product administration compared with the Reference product (Figure 3 and Figure 4). The observed difference between the products was statistically highly significant (*p* < 0.0001).

Thus, the nitrogen balance was better after the Test product than after the Reference product, indicating that the PT™ coating employed in the Test product increased the utilization of AAs and reduced their oxidation (Figure 3).

### 3.4. Total Quantities of Nitrogen from the AUC_0–300_ Contained in AAs and in BUN

The total AUC_0–300_ of AAs and BUN after the intake of the Test and Reference products from baseline until 300 min after dose intake and the differences in mol/L of the Test product minus the Reference product (Delta) were calculated (Table 4 and Table 5). To calculate the nitrogen content of circulating AAs, it was considered that individual AAs may contain one, two, or three nitrogen atoms. Starting from the AUC_0–300_ of each individual AA, the difference between the Test and Reference product was calculated. Starting from the contribution (in %) of each AA in the total AA AUC_0–300_ difference (Delta), it was possible to calculate a conversion factor for each subject (within product). There was a difference of 0.098 mol of plasma nitrogen contained in free AAs per litre in the 300 min after dosing between the Test product and the Reference product (Table 4).

To calculate the nitrogen differences of AUC_0–300_ represented by BUN between the Test and Reference products, it was considered that one molecule of urea contains two nitrogen atoms. There was a difference of 0.452 mol of plasma nitrogen present as BUN per litre until 300 min between the Test product and the Reference product (Table 5).

The Delta nitrogen AUC_0–300_ min (mol/L) contained in free AAs was −0.098 mol/L (geometric mean ratio 0.923, 90% CI 0.891–0.957) between the products, indicating that the nitrogen contents of AAs in plasma were rather similar after both products (Test and Reference). In contrast, the Delta nitrogen AUC_0–300_ min (mol/L) present as BUN was −0.452 mol/L between the Test and Reference products with an associated *p*-value of <0.0001, indicating that the administration of the Reference product was associated with more AAs being metabolized to urea than the administration of the Test product.

## 4. Discussion

The Test product was previously reported to be bioequivalent with the Reference product for all subgroups of AAs [18]. However, peak concentrations (C_max_) in plasma were significantly lower for all subgroups of AAs, indicating a delayed absorption of the Test product. In addition, BUN and the excretion of urea into the urine were significantly lower after the Test product compared with the Reference product. The present analyses indicate that the production of urea in proportion to systemic AA availability was significantly smaller after the administration of the Test product compared with the Reference product. This result supports the hypothesis that the Test product, manufactured using PT™, conferred the increased utilization of AAs for protein synthesis and reduced their oxidation and conversion to urea. The present analysis allowed to delineate how nitrogen balance is affected by the absorption kinetics of the ingested free AAs. The impact of the delayed absorption became most evident starting from 90 min after product ingestion and provided further support for the prolonged release obtained with PT™. Less wasted nitrogen means higher efficiency in the utilization of the AAs administered with the PT™.

Among experts caring for those with PKU, there is a consensus that the traditional phenylalanine-free AA formulations possess kinetic properties that lead to a suboptimal absorption. Furthermore, as children with PKU increase in age, adherence with dietary therapy commonly declines, particularly during adolescence and early adulthood [19,20,21]. Poor adherence with nutritionally supplemented AAs leads to both elevated blood phenylalanine concentrations and some nutritional deficiencies [22,23,24]. The distribution of the intake of AA mixtures evenly over the day and preferably together with or after meals [25] may have positive effects on blood phenylalanine levels as well as phenylalanine tolerance. However, from a behavioural point of view, this relentless routine may be a further challenge for adherence.

It is conceivable that the observed effects of PT™ on the disposition of free AAs, i.e., delayed and prolonged absorption, less oxidation, and thereby more efficient utilization compared with regular AA supplements, may be associated with clinically meaningful health benefits in terms of physiological body composition, better growth, and a more balanced supply of the ingredients of the AA mixture necessary for a successful treatment of subjects with PKU. The healthy subjects of the study were a limitation; a further investigation in patients with PKU is warranted.

## Figures and Tables

**Table 1:** Plasma amino acid and nitrogen concentrations after intake of the Test product and the Reference product.

Time	Test Product (AA, mmol/L) Mean (SD)	Reference Product (AA, mmol/L) Mean (SD)	Delta + AA Test vs. Reference (mmol/L) Mean (SD)	Delta ^‡^ Nitrogen Test vs. Reference (mmol/L) Mean (SD)
Baseline *	2.30 (0.30)	2.34 (0.26)	−0.05 (0.31)	−0.08 (0.43)
15 min	2.52 (0.33)	3.00 (0.46)	−0.43 (0.44)	−0.59 (0.60)
30 min	3.05 (0.44)	3.76 (0.59)	−0.65 (0.55)	−0.84 (0.72)
45 min	3.33 (0.48)	4.18 (0.61)	−0.80 (0.56)	−1.01 (0.76)
60 min	3.43 (0.48)	4.21 (0.54)	−0.80 (0.41)	−0.99 (0.58)
75 min	3.33 (0.48)	4.37 (0.64)	−1.06 (0.60)	−1.35 (0.82)
90 min	3.22 (0.44)	3.95 (0.55)	−0.77 (0.58)	−0.97 (0.81)
120 min	3.02 (0.43)	3.33 (0.37)	−0.35 (0.53)	−0.43 (0.73)
150 min	2.82 (0.38)	2.90 (0.31)	−0.10 (0.42)	−0.11 (0.58)
180 min	2.67 (0.38)	2.59 (0.26)	0.06 (0.35)	0.07 (0.49)
240 min	2.48 (0.30)	2.40 (0.24)	0.08 (0.33)	0.10 (0.48)
300 min	2.33 (0.29)	2.26 (0.23)	0.07 (0.29)	0.09 (0.40)

* Mean of concentration 30 min before and immediately before the intake of the products; + mmol/L AAs Test product minus mmol/L AAs Reference product; ‡ mmol/L total plasma nitrogen Test product minus mmol/L total plasma nitrogen Reference product. AA, amino acid; SD, standard deviation. AA concentrations in plasma and, consequently, amounts of nitrogen contained in plasma AAs were lower after the Test product than after the Reference product between 15 and 150 min after product intake and higher between 180 and 240 min, confirming the capacity of PT™ to slow down the absorption of free AAs.

**Table 2:** Blood urea nitrogen concentrations after the intake of the Test product and the Reference product and Delta nitrogen content.

	Concentration BUN (mmol/L)			
Time	Test Product Mean (SD)	Reference Product Mean (SD)	Delta BUN * (mmol/L) Mean (SD)	Delta Nitrogen ** (mmol/L) Mean (SD)
Baseline	3.89 (0.72)	4.08 (0.87)	−0.25 (0.56)	−0.49 (1.12)
15 min	3.82 (0.68)	4.16 (0.86)	−0.37 (0.56)	−0.74 (1.11)
30 min	3.94 (0.69)	4.30 (0.86)	−0.40 (0.86)	−0.79 (1.72)
45 min	4.04 (0.75)	4.57 (0.95)	−0.54 (0.58)	−1.07 (1.16)
60 min	4.15 (0.73)	4.77 (0.93)	−0.65 (0.57)	−1.29 (1.14)
75 min	4.28 (0.66)	5.08 (0.92)	−0.82 (0.58)	−1.64 (1.15)
90 min	4.37 (0.66)	5.26 (0.92)	−0.93 (0.65)	−1.85 (1.31)
120 min	4.50 (0.63)	5.47 (0.93)	−0.99 (0.62)	−1.99 (1.24)
150 min	4.58 (0.62)	5.45 (0.89)	−0.91 (0.65)	−1.83 (1.29)
180 min	4.55 (0.66)	5.45 (0.89)	−0.92 (0.76)	−1.84 (1.51)
240 min	4.58 (0.64)	5.26 (0.89)	−0.73 (0.64)	−1.45 (1.28)
300 min	4.53 (0.63)	5.10 (0.82)	−0.60 (0.63)	−1.21 (1.24)

* mmol/L BUN Test product minus mmol/L BUN Reference product; ** Delta mmol/L nitrogen = Delta mmol/L BUN × 2; BUN, blood urea nitrogen; SD, standard deviation.

**Table 3:** Comparison of Delta nitrogen contained in amino acids with Delta nitrogen as blood urea nitrogen *.

Time	Delta Nitrogen in AAs (mmol/L) Mean (SD)	Delta Nitrogen in BUN (mmol/L) Mean (SD)	Delta Nitrogen in AAs Minus Delta Nitrogen in BUN (mmol/L) Mean (SD)
Baseline	−0.08 (0.43)	−0.49 (1.12)	0.42 (1.07)
15 min	−0.59 (0.60)	−0.74 (1.11)	0.15 (0.93)
30 min	−0.84 (0.72)	−0.79 (1.72)	−0.05 (1.66)
45 min	−1.01 (0.76)	−1.07 (1.16)	0.11 (1.23)
60 min	−0.99 (0.58)	−1.29 (1.14)	0.30 (1.26)
75 min	−1.35 (0.82)	−1.64 (1.15)	0.29 (1.27)
90 min	−0.97 (0.81)	−1.85 (1.31)	0.88 (1.39)
120 min	−0.43 (0.73)	−1.99 (1.24)	1.56 (1.43)
150 min	−0.11 (0.58)	−1.83 (1.29)	1.72 (1.35)
180 min	0.07 (0.49)	−1.84 (1.51)	1.92 (1.55)
240 min	0.10 (0.48)	−1.45 (1.28)	1.55 (1.28)
300 min	0.09 (0.40)	−1.21 (1.25)	1.30 (1.24)

* Delta refers to total nitrogen in AAs or BUN after ingestion of the Test product minus the total nitrogen in AAs or BUN after ingestion of the Reference product. AA, amino acids; BUN, blood urea nitrogen; SD, standard deviation.

**Table 4:** Comparison of total plasma amino acids AUC_0–300_ and related quantity of nitrogen after administration of the Test and Reference product.

	Mean AAs AUC_0–300_ (mol/L/300 min) Mean (SD)	Quantity of Nitrogen (mol/L/300 min) Mean (SD)
Reference product	0.9146 (0.075)	1.302 (0.100)
Test product	0.8391 (0.099)	1.209 (0.140)
Difference Test–Reference	−0.078 (0.096)	−0.098 (0.135)

AA, amino acid; AUC_0–300_, area under the concentration–time curve from 0 to 300 min; SD, standard deviation.

**Table 5:** Comparison of total nitrogen present as BUN AUC_0–300_ after the Test and Reference products.

	Mean BUN AUC_0–300 min_ (mol/L/300 min) Mean (SD)	Quantity of Nitrogen (mol/L/300 min) Mean (SD)
Reference product	1.5729 (0.266)	3.146 (0.532)
Test product	1.3574 (0.201)	2.715 (0.402)
Difference Test–Reference	−0.226 (0.187)	−0.452 (0.375)

AUC_0–300_, area under the concentration–time curve from 0 to 300 min; BUN, blood urea nitrogen; SD, standard deviation.
